# Insights into health care seeking behaviour for children in communities in KwaZulu-Natal, South Africa

**DOI:** 10.4102/phcfm.v9i1.1378

**Published:** 2017-05-29

**Authors:** Lyn Haskins, Merridy Grant, Sifiso Phakathi, Aurene Wilford, Ngcwalisa Jama, Christiane Horwood

**Affiliations:** 1 Centre for Rural Health, University of KwaZulu-Natal, South Africa

## Abstract

**Background:**

South African infant and child mortality remains high, with many deaths occurring outside the formal health services. Delayed health care seeking represents a large proportion of these deaths.

**Aim:**

To generate knowledge about the role of, and influences on, caregivers with regard to decision-making about when and where to seek care for sick children.

**Setting:**

Two communities in KwaZulu-Natal.

**Methods:**

A qualitative, exploratory design employing participatory research techniques was used to undertake focus group discussions with community members.

**Results:**

Health care seeking for a sick child was described as a complex process influenced by multiple carers using multiple providers. Decision-making about seeking health care for a sick child was not an individual effort, but was shared with others in the household and guided by how the symptoms were perceived, either a Western illness or African illness. A sick child could either be treated at home or be taken to a variety of places including clinics, private doctors, traditional healers, faith healers and hospitals. Traditional healers were associated with the treatment of illnesses perceived to be traditional. Few participants said that they would take their child back to the original health provider if the child remained ill, but would move from one provider to another until the child’s health improved.

**Conclusion:**

The formal health system needs to ensure that sick children are identified and managed appropriately to reduce child deaths. Knowledge and understanding of health care seeking behaviour for sick children by carers is an important aspect. Interventions need to be designed with these contextual issues in mind.

## Introduction

Child mortality remains an urgent global health concern despite substantial gains in recent years. In 2015, 5.9 million children aged less than 5 years died globally; including over three million children in sub-Saharan Africa, where one in 12 children die before their fifth birthday.^1^ Most deaths are because of conditions such as diarrheal disease, pneumonia, malaria, measles, HIV or AIDS and malnutrition, which are treatable and preventable with available, cost-effective interventions.^2,3^ However, substantial inequity remains between low- and high-income countries. There are complex reasons for this, and it is therefore important to take a holistic approach to addressing inequity in child mortality.

Infant and child mortality in South Africa remains unacceptably high, higher than the rate in other middle-income countries,^4^ despite the availability of free health care for mothers and children. Child mortality data are frequently incomplete and unreliable, but by using three important data sources (vital registration data, district health information system and child problem identification programme), it is estimated that over 50% of child deaths occur outside the formal health service, with such deaths occurring mostly at home and often amongst children who have not been seen by a health worker.^5^ Data from KwaZulu-Natal province (KZN) suggest that almost 60% of child deaths occur at home, and such deaths are more common in urban areas compared with rural areas.^5^

Severe illness resulting from delayed health care seeking represents a large proportion of the mortality burden in South Africa.^3^ Appropriate health care seeking behaviours can reduce the occurrence of severe and life-threatening childhood illnesses.^6^ However, in African settings, health care is still a combination of different systems: biomedical care, self-medication and traditional healers, with traditional health care being the principal type of health care in many African communities.^7^ The traditional health care sector is a very large and traditional concepts of health and healing running alongside the biomedical model with a high demand for traditional medicine, even in urban areas.^8^

### Theoretical framework

There are multiple theories and models that explain individuals’ health care seeking behaviour, of which Andersen’s model of health care utilisation is one.^9,10^ Andersen suggests three categories of determinants for health care seeking: predisposing characteristics, enabling characteristics and need-based characteristics.

Firstly, predisposing characteristics represent the individual’s tendency to utilise health care services; an individual is more or less likely to use health services based on demographics, position within the social structure and beliefs of health services benefits. Secondly, enabling characteristics include resources available within the family and the community, including economic status and the location of residence, as well as access to health care facilities and the availability of persons for assistance. Thirdly, need-based characteristics include the perception of the need for health services, whether they are individual, social, or clinically evaluated perceptions of need. This model shows that a complex interaction of individual, household and community factors exists during health care seeking, and such complex dealings are likely to result in delayed care for young children.

Early identification of severe illness at the household level and timeous access to evidence-based interventions is important for achieving further improvements to child mortality, particularly in vulnerable and underserved communities. Therefore, it is important to understand the ways in which communities seek health care for ill children, and the factors that influence health care seeking behaviour by caregivers of young children in order to design appropriate interventions to improve health care seeking. In this paper, we present the findings of a qualitative study to generate knowledge about the role of, and influences on, caregivers with regard to decision-making about when and where to seek care for their child.

## Methods

### Study design

We used an exploratory and descriptive qualitative design^11^ in which focus group discussions (FGDs) were conducted, utilising participatory research (PR) techniques to explore the knowledge, roles and factors that influence care givers’ decisions and practices about when and where to seek care for sick children. The research conducted was not a comprehensive participatory process but rather it employed PR techniques to access the local knowledge of participants.

### Study setting

Two sites, one rural and one peri-urban, were chosen in one district of KZN, South Africa, based on our ability to access community health workers (CHWs) via a non-governmental organisation (NGO) working with families in the area. CHWs were then employed as key informants to assist with selection of appropriate participants.

### Study population and sampling

This study used purposive sampling to select participants with children aged less than 5 years living in their households. Participants were sampled according to age and gender and included young women (18–34 years), young men (18–34 years), older women (> 35 years) and older men (> 35 years). Entry into the communities and households was facilitated by CHWs. CHW approached community members whom they knew, from visiting their homes, to participate in the FGDs.

### Data collection

A FGD guide was developed in consultation with experts in child health and piloted amongst women in the district in partnership with a local NGO, and relevant changes made.

**FIGURE 1:**
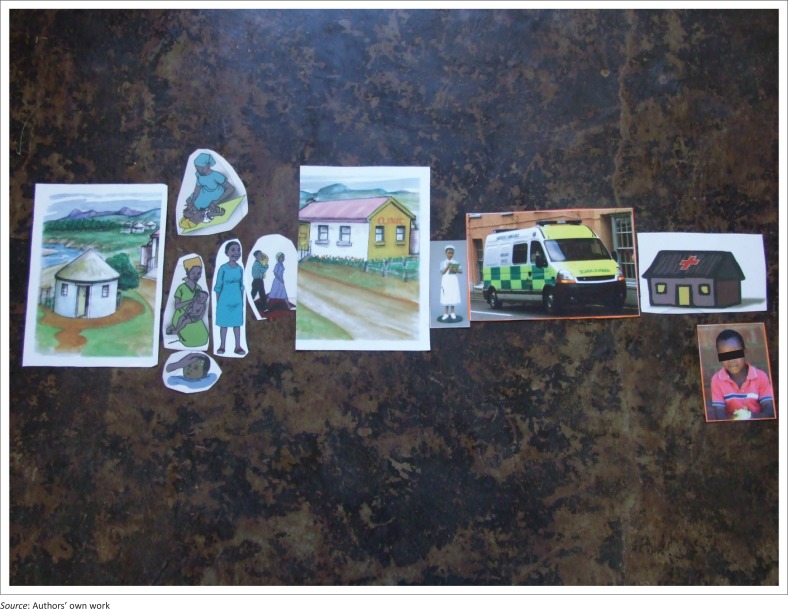
Examples of pictures given to the participants for the participatory research activity.

**FIGURE 2:**
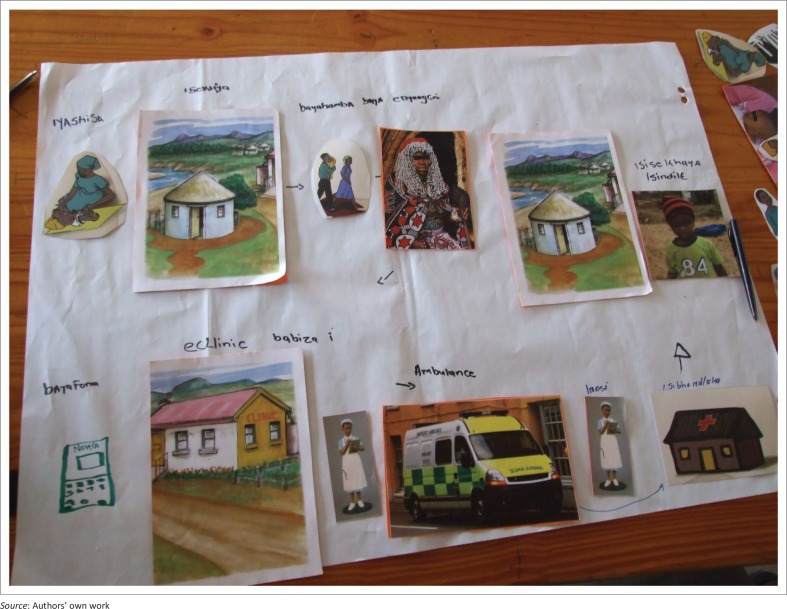
An example of the story told by a group.

A PR technique was used within the focus group process. These techniques are valuable as they maximise participation and facilitate the expression of multiple voices within the research process.^12^ During FGDs, participants were verbally given three different scenarios (Table 1) of sick or at risk children and asked to discuss each scenario in relation to the care seeking practices that they would adopt in these situations. The scenarios served as a prompt and encouragement for the participants to speak freely about their experiences. Scenarios were based on important health issues prevalent in the South African context that would be expected to be common in the community. Participants were asked to use pictures depicting different people and places (Figure 1) to build the story of the people and places they would go to access care for the sick child in each scenario. Instructions to participants were that the pictures could represent different people and places in their community where they seek help for children and could be anyone or any place they wanted them to be. Working in groups, participants were asked to tell the story of a child and how the child’s parents or caregiver went about seeking help for the child. They did not have to use all the pictures. Participants were given 15 minutes to complete the activity and a further 20 minutes for discussion of the picture they had produced (Figure 2).

**TABLE 1:** Scenarios provided to focus group discussions participants.

Scenario	Description
Scenario 1: Coughing child	We are now going to talk about what caregivers in your community do when a child is ill. Here is a baby who is 8 months old who is coughing. After the first day of coughing, the baby became more ill, had a fever and could not breathe well. The caregiver became more worried. Think of the kinds of places or people you or others would take this child to get help when they are sick.
Scenario 2: Weight loss	This child is 14 months old and has lost weight: Should the parent or caregiver be worried? When might they worry? What are some of the reasons for a child losing weight? Should the caregiver go to someone for help? Who or where might she or he go to?
Scenario 3: Delayed milestone	This child was walking but now is only crawling. Should the parent or caregiver be worried? When might they worry? What are some of the reasons for a child to regress from walking to crawling? Should the caregiver go to someone for help? Who or where might she or he go to?

The FGDs were undertaken by experienced qualitative researchers (one female, one male) in easily accessible community halls and a local school. FGDs were conducted in the local language (isiZulu) and lasted for approximately 2 hours.

All FGDs were audio-recorded, transcribed verbatim and translated into English for analysis.

The researchers engaged in a process of reflexivity throughout the study. They constantly reflected on their role as researchers (assumptions and preconceptions) and their role in the research relationship (power dynamics). Qualitative research is an interactive meaning-making process, and thus, reflexivity on the entire research experience is required. The researchers conducted a pilot focus group where they identified questions that needed to be revised based on reflexive practice. Close attention was also paid to the power dynamics in the focus groups. The researchers’ position was also constantly acknowledged and reflected upon in the analysis process.

### Data analysis

The data from the FGDs were analysed using an inductive approach and standard guidelines were used for thematic coding as the primary analytic strategy.^13^ All transcripts were entered into qualitative data analysis software (NVivo version 9). Members of the analysis team, comprising three experienced qualitative researchers (one male and two female), collaborated to develop a codebook of themes around the main topics until saturation was reached and no new themes or topic areas emerged from reading unique transcripts. To ensure inter-rater consistency, the analysis team compared and discussed their coding of transcripts. The analysis focused on exploring the care seeking decisions and practices of caregivers. The analysis team worked together closely to resolve any interpretation discrepancies.

Validity in qualitative research is determined by the extent to which the findings accurately represent the data collected.^14^ In this study, validity was ensured through consistency of findings, accurate representation of all relevant reviews through checking for deviant cases in the data and through adequate and systematic use of the original data, for example using quotations from various FGDs rather than concentrating on one source.

### Ethical consideration

All participants gave their verbal informed consent in their local language before the beginning of the focus group discussions. Participants were assured that all contributions would remain anonymous, and no identifying information was collected. All audio recordings and transcripts were kept in password-protected computer files at the Centre for Rural Health. Ethical approval was obtained from the Human and Social Science ethics committee at the University of KwaZulu-Natal (Reference number: HSS/0161/10).

## Results

Fifteen FGDs were conducted with a total of 84 participants during June–August 2010. Four FGDs were conducted with young women (aged 18–34), three FGDs with young men (aged 18–34), three FGDs with older women (> 35 years) and one FGD with older men (> 35 years). In addition, three FGDs were conducted with women of mixed ages and one FGD with men of mixed ages. Participants in FGDs are shown in Table 2.

**TABLE 2:** Participants in focus group discussions.

Variable	*N* = 84	%
Women aged between 18 and 35 years	39	46.4
Women older than 35 years	21	25.0
Men aged between 18 and 35 years	13	15.5
Men older than 35 years	11	13.1
Participants from rural community	44	52.4
Participants from peri-urban community	40	47.6
Participants unemployed	73	87.0
Participants employed	11	13.0

### Decision-making about health care seeking for a sick child

Health care seeking for a sick child was described as a complex process influenced by multiple carers and the use of multiple providers. Carers’ perceptions of illness differed according to the type of carer making the decision and the symptoms of the child. Overall, decision-making about seeking health care for a sick child was not perceived as an individual effort, but rather something that was shared with others in the household.

‘We’d discuss it together, … perhaps you normally talk to an adult, the man of the house, perhaps a grandfather or the grandmother, talk to them and grandma will say “let us give him this first or go and ask for this from so and so, if we can’t help him then we will go the clinic.”’ (FGD 4, young, woman)

Most respondents agreed that it was often the mother who first identified that the child was ill and then consulted others in the household to decide on a course of action. Mothers were identified as decision-makers for health care seeking, as they were believed to have a maternal instinct and could understand the child better than others, and were said to be educated on the child’s health by clinic staff.

‘The mother’s biggest role is that one; because you are the one that sees everything about the child. Even if someone else sees it, you would have already seen that the child is not well.’ (FGD 6, mixed age group, woman)

Parents were also identified as joint decision-makers by a few participants.

‘Eh the mother has influence; I [*the father*] also have an influence; I would say we both decide.’ (FGD 1, older, man)

Grandmothers were viewed as having a prominent role to play in the care of young children and as key role-players when a child was ill. Respondents identified grandmothers as being very knowledgeable about traditional ways including what herbs to use and how to administer enemas. One young mother stated:

‘If a child is sick I take him to the doctor; she [*the grandmother*] gets him herbs.’ (FGD 4, young, woman)

Grandmothers had a significant influence on health care seeking practices, with the mother often consulting the grandmother when faced with making a decision about a sick child.

*‘*Like when the child is ill, there are things that the grandmother could do that could assist the child. For example, the grandmother can help with the traditional medicine for an enema; the child does not necessarily have to go to the clinic or see the doctor.’ (FGD 2, young, woman)

The mother and grandmother were often identified as making decisions jointly.

‘Most of the time it is the mother and the grandmother who know that this illness would be treated by this or that.’ (FGD 2, young, woman)

In general, fathers were reported to have a lesser role in health care seeking, particularly for younger children. However, men and women perceived the role of the father differently. Men reported themselves as having a greater role in child care compared with the women’s perception of men’s role. Women expressed that men had a minimal role in the care of children, and many women spoke disparagingly about men’s involvement. A male respondent discussed the following:

‘Most fathers disappear as soon as the child is born, and that child becomes the responsibility of the mother’s family.’ (FGD 8, mixed age group, man)

However, in some cases, respondents cited the father as having an influence in decision-making regarding an ill child. One respondent stated:

‘The father also has an influence; if a child is not well he will say “mother of my child, take the child to the clinic.”’ (FGD 6, mixed age group, woman)

Despite this, it was reported that when the child is taken to clinic or hospital the father has to be notified. When asked why this was so, women said that a father would blame a mother if the child died and he was unaware of the child’s illness. In some cases, fathers were called upon to provide financial support to pay for a clinic visit. However, in such cases the mother reported that she would tell the father that she needed money to go to the clinic, so that the decision was already made by the mother.

It was also mentioned that many family members could be involved in a decision about health care seeking practices. One respondent stated that she would consult ‘the family, because it might happen that one doesn’t have a grandmother. … You tell whoever is there at the time.’ (FGD 5, mixed age group, woman)

Another participant said that if an older person was not available at home, she would consult an older, more knowledgeable neighbour.

### Perceptions of child’s symptoms: Traditional versus Western

An important factor influencing health care seeking behaviour was the symptoms of the child and how these were perceived by the carer. The three illnesses in our scenarios (Figure 1) were perceived differently either as Western type illness or traditional illness or a mixture of both.

Cough-related illnesses, such as asthma, flu or tuberculosis (TB), were generally perceived as Western illnesses.

‘When the child finishes the clinic prescription but the cough does not stop, then something is more serious. It might be, that at home, there is someone with TB; then the clinic would tell you to take the child to the hospital for a check-up.’ (FGD 7, mixed age group, woman)

However, *isela* was also associated with coughing by a few participants, and in this case traditional methods were used. *Isela* refers to sores on (usually on the anus) or inside a child, treated by *goqoza*, which is the process of inserting toothpaste into the child’s anus. One respondent stated:

‘When the child starts coughing it could be a sign for *isela*; so you have to give an enema to the child.’ (FGD 11, young, woman)

The second scenario was about a child losing weight. Weight loss was mainly associated with Western perceptions of illness, and most respondents felt that weight loss was either the result of poor diet or an illness such as worms or TB and said that they would take their child to the clinic if they had lost weight. One participant suggested that: ‘the reason for weight loss could be that the child is not fed adequately.’ (FGD 11, young, women). However, some respondents suggested that traditional reasons could be the cause; one respondent stated:

‘You can also go to the traditional doctors maybe they will tell you why the child is losing weight, it could be traditional.’ (FGD 2, young, woman)

Regressing from walking to crawling (scenario 3) was viewed by many participants as a traditional illness and certain things had to be done to protect the child. Participants discussed that it may be as a result of the ancestors being unhappy or it could be because of bewitchment, and herbs should be burned for the child for protection.

‘There are evil things that people do, that may cause the child to return to the crawling stage.

They [*traditional healers*] will tell you what is wrong. … Then you come this side [*to the clinic*]; here they will find what is lacking in the child, perhaps vitamins, and they provide the child with boosters.’ (FGD 1, older, man)

Some participants felt that the reason for the child’s lack of development could be traditional issues that needed to be addressed by the family.

‘The child could return to crawling stage because of the traditional issues. For example, my child never had a problem, he was growing very fast, but all of the sudden the child stopped growing. Then I was very concerned and I asked myself what is wrong? My child was growing very fast in everything; he learnt to stand when he was nine months old and remained at that stage until he was one year six months. He was talking and doing everything but there was no movement. He could not walk. I realised something was wrong, then I took him to his family and they helped me. [*Laughter*] … they did their traditional ritual then he started walking.’ (FGD 7, mixed age group, woman)

A few participants hinted at HIV being the cause, by mentioning ‘the virus’, but did not say this explicitly. Other reasons suggested for regressing from walking to crawling were muscle- or bone-related diseases, malnutrition, sexual abuse and not spacing pregnancies adequately.

### Places for health care seeking

Participants reported that a sick child could either be treated initially at home or taken to a variety of places including clinics, private doctors, traditional healers, faith healers and hospitals (see Figure 2 for an example of a story told by participants). Very few participants said that they would take their child back to the original health provider if the child continued to be ill, but would rather move from one provider to the next until the child’s health improved. This was common to all three scenarios. There was some reference to carers returning to a provider if they were specifically told to return at a particular time if the child’s health did not improve. In the extract below the respondent explains that you try everything to ensure the health of your child.

‘When you have a sick family member you don’t stand at one place, because if you have money you don’t consult one doctor. You take him to this doctor and see that he is not getting better, you move on to the next doctor and see how he deals with it; you see that? You as well, will go out to other people to see if you can get a different view that can help you and your child.’ (FGD 5, mixed age group, woman)

### Home care

Carers frequently reported home treatment as the starting point when a child became unwell, including medicines from the clinic or pharmacy and traditional remedies. Home treatment was frequently reported as the first point of care for children with a cough. Participants spoke of treating the child using cough medicine (cough mixture) or pain killers such as paracetamol from the pharmacy or clinic; they also referred to using castor oil to treat the coughing child. One participant explained:

‘At the clinic they recommend you must always keep medicine in case your child falls ill … the child should drink that medicine, and if they notice that it continues then they should take the child to the clinic.’ (FGD 15, young, man)

Many respondents spoke of first using traditional home remedies before getting further help. If the cause of the illness was seen as related to traditional illness, traditional home remedies could be administered in the form of traditional herbs which may be burned, made into a drink, or given as enemas with herbs or toothpaste.

‘That is why we burn herbs for the baby … we fight things. You have to burn herbs for the baby so that the baby can be protected.’ (FGD 1, older, man)

### Western health services: Clinics and private doctors

Clinics were generally the first point of care for illnesses regarded as a Western illness. A few participants said that they would take the child directly to the clinic as a first response, but most said that they would do so if the child did not improve. One participant explained that she would ‘wait at least three days, and if you see that the herbs do not work, then you would go to the clinic.’ (FGD 12, young, woman)

Reasons given by participants for taking a child to a clinic included: if the illness was beyond the carer, availability of medicine and trained health professionals at the clinic, if traditional medicine did not work and if the child’s health was not improving.

‘You should go to the clinic, because at the clinic there are educated people, who are educated about human life, because there you find nursing sisters and they check your child and see that when this is like this it needs a doctor. Like we said earlier, they will refer you to the hospital.’ (FGD 5, mixed age group, woman)

Hospitals were only mentioned in the case of severe illness, and usually as referrals from the clinic, although some participants did say that they would go directly to the hospital. The main reasons for attending the hospital was if the child’s health had not improved or to gain access to resources not available at clinics, for example X-ray facilities, oxygen, highly trained staff, doctors and medicine. Participants also spoke about calling the ambulance to go to the hospital if the child was very ill.

Private doctors were viewed as expensive and very few participants mentioned that they would go directly to the doctor for help. Others said they would first go to the clinic and, if the child’s health did not improve, they would then go to the doctor. One participant said that they would first go to the traditional healer and then move on to the private doctor if the child did not get better. Reasons for going to the doctor included the child not getting better, and if one could afford the service.

Community health workers were rarely mentioned; only one respondent said that they would consult a CHW:

‘The community caregiver touches and examines to check if the baby is really ill. And she said no this baby is ill. Then she said the baby must be taken to the clinic.’ (FGD 1, older, man)

### Traditional health services

Participants did not immediately speak of the use of traditional healers but, as discussions progressed, they started to reveal that they were used. Traditional healers were generally associated with the treatment of traditional illnesses. Many carers acknowledged that enemas are frowned upon by Western medicine but people in the communities still continued to believe in them. Participants highlighted the fact that the clinic may not be able to treat some illnesses.

‘They [*traditional healers*] assist with things that the clinic is unable to treat.’ (FGD 1, older, man)

Overall, reasons for going to traditional healers were if the child’s condition was not improving, or if the illness was considered a Zulu or African illness. Many participants identified traditional healers being able to tell you what the real cause (traditional cause) of an illness is ‘usually the traditional doctor has that gift of telling you what might be the problem, you see.’ (FGD 8, mixed age group, man).

Participants also spoke of bewitchment. One participant explained that ‘there are also *muthi* inflicted illnesses such as “*meqo*” [*Illness that you pick up by walking over it*].’ (FGD 3, older, woman).

However, some respondents discussed how traditional care was sometimes ineffective:

‘It’s just that their method delays; it take forever, while on the other [*hand*] the illness is worsening. Sometimes you find that they are just guessing, foretelling lies, you see, while the illness is worsening. At the clinic you can quickly get help because there are doctors.’ (FGD 1, older, man)

A few participants also mentioned that they could go to faith healers for assistance.

‘Yes, because we are [*black*] people. When I come back from the clinic they will give me stuff and advise me that I should buy him things that give him energy. If he runs out of energy they give you proper things that you should try. If that fails, then I will wake up the following day and go to the faith healer, perhaps I will get help.’ (FGD 3, older, woman)

## Discussion

This study found that recognising symptoms in sick children and deciding on an appropriate pathway for treatment is a complex, collaborative, communal process, involving household and community members, as well as primary caregivers, to varying degrees. The results support other studies conducted on determinants of health seeking behaviour as highlighted by the Anderson model,^9,10^ stating that family beliefs and decision-making are important factors that influence health care seeking for young children. When grandmothers were present, they usually contributed to or made the final decisions on where to seek care first, because they were considered knowledgeable and experienced. This does not mean mothers’ opinions were ignored. Mothers were regarded as having maternal intuition and often identified the symptoms of illness first. Mothers’ decisions to seek care are largely influenced by changes in the child’s behaviour and evidence of presenting symptoms.^15^ Participants felt that grandmothers were generally the final decision-makers on whether a child went to a traditional healer when sick^8^ or whether Western services were sought. The role fathers played in care seeking was contested, with fathers feeling that they played a greater role in the child’s life and health compared to the opinions of mothers.

We concur with Colvin et al.^16^ that efforts to provide care for sick children hinge on the family’s social, cultural and religious beliefs about the causes of the illness and acceptability of the interventions. Thus, people viewed illnesses through variable lenses; their perceptions informed whether they sought help from traditional healers or Western health care services. This study found that such service providers were used interchangeably and multiple service providers were used to treat one illness. The way carers understood the reason and cause for such illnesses determined the pathways of care chosen.

We found that illness was identified as either Western or traditional, and the boundaries between the two were blurred. However, home-based care was usually the first point of care^17^ whichever treatment pathway was chosen, with some carers giving medicine from the pharmacy first and some favouring the use of traditional medicines. Grandmothers often administered the traditional medicine, preparing the necessary herbs or administering the toothpaste enemas. The demand for traditional medicine is said to be high, even in urban areas,^8^ and many carers acknowledged that, although enemas were frowned upon by Western medicine, they continued to believe in them. However, home-based care could lead to delays in appropriate health care seeking and put children at risk. Delayed health care seeking is an important cause of mortality, and children with a prolonged illness where symptoms do not resolve with first line treatment are particularly vulnerable to morbidity and mortality.^18^

Community health workers are members of the community and are regularly present in the home environments. They should be aware of the traditional beliefs around childhood illness and be able to proactively address this issue during consultations with mother and caregivers. Mothers and caregivers in the focus groups were reluctant to mention treatments given by traditional healers, possibly because they feared being scolded or they thought that Western medicine practitioners would not consider traditional treatments as relevant. CHWs have an important role to play in assisting families with when and where to seek care for their children and can educate family members and mothers on recognising danger signs in children, which may allow for timely access to health care.

In all the scenarios presented very few participants said that they would take their child back to the original health provider if the child continued to be ill. They would rather move from one provider to the next until the child’s health improved. This pattern of health care seeking is problematic as the severity of the illness may not be understood because of constantly changing the health provider. Messages given by health workers should emphasise that if there is no improvement the child should to be brought back to the clinic. This is an important message within the integrated management of childhood illness (IMCI) treatment guidelines used in by health workers in clinics.^19^ CHWs should also reinforce such messages in households if a child’s symptoms do not resolve.

Given that grandmothers are key decision-makers and traditional healers key providers of care, we need to question what health messages should be given to caregivers, who the messages should target and what needs to be addressed in the message. Grant et al.^20^ describe how health workers navigate the complex terrain of providing Western health care within the context of coexistence of traditional medicine, and advocate for integrated holistic care in which traditional care is acknowledged and managed. Health messages for caregivers must similarly acknowledge non-harmful traditional practices but also target community health messages that promote safe practices and health care seeking behaviour. As family structures vary and decisions are not made individually, a family-centred approach should be taken when giving messages about appropriate care seeking. CHWs in communities are an important link for the chain of care, and can bridge the gap between Western and traditional health care as being both community members and health practitioners at the household level.

Integrated management of childhood illness is the approach taken to manage sick children at primary health care (PHC) facilities in South Africa. IMCI approach recognises the role of families in a child’s health care by providing advice for home care for sick children when CHW visit the home in the community as advocated in the community component of IMCI.^21^ This includes understanding how symptoms and illnesses are classified locally, as well as educating families about when children need to receive immediate medical attention.^8^ It is important to empower communities to explore and address issues that facilitate or hinder key family practices for effective child health care. Health education campaigns should pay attention to the symptoms that mothers themselves recognise as important.^8^

### Strengths and limitations

We used a unique PR technique with pictures and scenarios during the FGDs, which allowed participants opportunities to participate freely without being bound by formality and cultural power dynamics. Another strength of the study was that we included the perspectives of men in the community to ensure all voices in the community were heard.

The limitation of this qualitative study is that it was localised to one predominantly rural district South African setting.

## Conclusion

For health care seeking practices to improve, it is important to understand the collective nature of decision-making in a family and the roles people play. Interventions need to be designed with this contextual understanding. As traditional healing methods are widely used and accepted, health care providers using a more biomedical approach to health care need to develop relationships with local traditional healers in order for both types of providers to have their place in health care provision without putting children at risk. CHWs could help in bridging the gap between traditional healers and clinics or private doctors.
